# Disordered eating in Sami and non-Sami Norwegian populations: the SAMINOR 2 Clinical Survey

**DOI:** 10.1017/S1368980017003597

**Published:** 2017-12-10

**Authors:** Kirsti Kvaløy, Marita Melhus, Anne Silviken, Magritt Brustad, Tore Sørlie, Ann Ragnhild Broderstad

**Affiliations:** 1 Centre for Sami Health Research, Department of Community Medicine, UiT The Arctic University of Norway, 9037 Tromsø, Norway; 2 HUNT Research Centre, Department of Public Health and Nursing, Faculty of Medicine and Health Sciences, NTNU – Norwegian University of Science and Technology, Trondheim, Norway; 3 Sámi Norwegian National Advisory Board on Mental Health and Substance Abuse (SANKS), Karasjok, Norway; 4 Department of Community Medicine, UiT The Arctic University of Norway, Tromsø, Norway; 5 Department of Clinical Medicine, UiT The Arctic University of Norway, Tromsø, Norway; 6 Department of Mental Health and Substance Abuse, University Hospital of North Norway, Tromsø, Norway; 7 Medical Department, University Hospital of North Norway, Harstad, Norway

**Keywords:** Disordered eating, Eating Disturbance Scale, Sami, Obesity, SAMINOR

## Abstract

**Objective:**

The present study aimed to investigate disordered eating (DE) among Sami compared with non-Sami residing in northern Norway.

**Design:**

In a cross-sectional design, stratified by sex and ethnicity, associations were tested between DE (Eating Disturbance Scale; EDS-5) and age, education level, BMI category, anxiety and depression, physical activity and consumption of snacks.

**Setting:**

The SAMINOR 2 Clinical Survey (2012–2014) based on the population of ten municipalities in northern Norway.

**Subjects:**

Adults aged 40–69 years; 1811 Sami (844 male, 967 female) compared with 2578 non-Sami (1180 male, 1398 female) individuals.

**Results:**

No overall significant ethnic difference in DE was identified, although comfort eating was reported more often by Sami individuals (*P*=0·01). Regardless of ethnicity and sex, symptoms of anxiety and depression were associated with DE (*P*<0·001). Furthermore, DE was more common at lower age and higher BMI values. Education levels were protectively associated with DE among Sami men (*P*=0·01). DE was associated (OR, 95% CI) with low physical activity in men in general and in non-Sami women (Sami men: 2·4, 1·4, 4·0; non-Sami men: 2·2, 1·4, 3·6; non-Sami women: 1·8, 1·2, 2·9) and so was the consumption of snacks (Sami men: 2·6, 1·3, 5·0; non-Sami men: 1·9, 1·1, 3·1; non-Sami women: 2·1, 1·3, 3·4).

**Conclusions:**

There were no significant differences regarding overall DE comparing Sami with non-Sami, although Sami more often reported comfort eating. There were significant sex and ethnic differences related to DE and physical activity, snacking and education level.

A growing societal acceptance of overweight and obesity exists along with the global obesity concern. Linked to a higher social status, however, being healthy, fit and thin is emphasized, with one consequence being an eating disorder (ED) prevalence increase^(^ ^1^ ^,^ ^2^ ^)^ balancing between healthy eating and eating pathologically healthy^(^ ^3^ ^)^. The lifetime prevalence in Western societies for the classically defined ED has been estimated to ~0·5% for anorexia nervosa (AN) and 1·0% for bulimia nervosa (BN), with three to eight times higher prevalence in women^(^ ^4^ ^,^ ^5^ ^)^. Overall, ED appear to be clearly associated with being female and younger^(^ ^6^ ^)^.

Binge eating disorder (BED), which was newly recognized in the *Diagnostic and Statistical Manual of Mental Disorders*, 5th edition, is characterized by recurrent episodes where eating large quantities of food is associated with the loss of control over eating and experiencing shame, distress or guilt afterwards^(^ ^7^ ^)^. Both BN and BED are increasing in prevalence globally and are associated with psychological and physical impairments, including overweight and obesity^(^ ^7^ ^,^ ^8^ ^)^. The relationship between binge eating and obesity is complex and likely bidirectional^(^ ^9^ ^)^.

Significant health risks due to physical and psychological co-morbidities linked to ED are seen and studies have shown that individuals with ED have higher risks of emotional and mental health problems especially pronounced in patients with BED and BN ^(^ ^10^ ^–^ ^12^ ^)^. A recent large clinical Swedish study which included 11 588 participants with various types of ED demonstrated that 71% suffered from at least one clinical or other adverse health condition, whereof the most common diagnosis was anxiety (53%)^(^ ^13^ ^)^. Earlier clinical and epidemiological studies have shown that depressive and bipolar diseases are the most common psychiatric co-morbidities among BED patients^(^ ^12^ ^)^.

Individuals with disordered eating (DE) show signs and symptoms of ED without reaching the clinical threshold for ED diagnosis^(^ ^14^ ^)^ and prevalence rates as high as 12% have been reported in Norwegian women previously^(^ ^15^ ^)^. The prevalence of both ED and DE is higher in females, although BED shows a lifetime prevalence closer to 1:1 in males and females (1·6 and 2·0%, respectively)^(^ ^16^ ^)^. In both sexes DE traits seem to be tracking from adolescence through to adulthood^(^ ^17^ ^)^, emphasizing the importance of early preventive measures. Factors influencing DE in males are mostly unknown although assumed to be linked to ideals related to appearance and performance (e.g. muscle dysmorphia)^(^ ^18^ ^)^.

Historically, ED have been thought to primarily affect Caucasian females in industrialized Western Europe and North America, and there are rather few studies based on data from non-Western countries. The culture and landscape of ED seem to be changing, however, and there is documentation of increased prevalence in a number of countries and cultures worldwide^(^ ^19^ ^)^. Social pressure resulting from the standards of female beauty imposed by modern Western culture is an important associated factor of DE, which also likely affects the increased rates of ED observed in non-Western countries^(^ ^20^ ^)^. Factors influencing body image and further risks of ED and DE are presumably both culturally and socially dependent, although ED initially described in Western Europe and North America were recognized to be a ‘culture-bound syndrome’. At present, the prevalence of AN appears relatively stable in North America and Western Europe, whereas the prevalence of BN may be decreasing among Caucasians, but increasing among Black Americans and Latinos in North America^(^ ^19^ ^)^. Recent expansion of studies based on various populations has further shown an increased prevalence of ED in general among individuals of diverse cultural and ethnic backgrounds across both Asia and the Arab region^(^ ^21^ ^,^ ^22^ ^)^.

The Sami are indigenous people traditionally living in northern parts of Norway, Finland, Sweden and the Kola Peninsula of Russia, where the majorities reside in Norway. The Sami people embrace a variety of languages, cultures and other differential social conditions and are very heterogeneous, with several different cultures depending on geographic area. Each area has its own characteristic features linked to location, climate, majority or minority status of the Sami in relation to the non-Sami population, and implementation of preservation measures for the Sami language and its various dialects^(^ ^23^ ^)^.

Data from the SAMINOR 1 Survey (2003–2004) based on 7301 men and 7841 women (aged 36–79 years) of both Sami and non-Sami origin (35% Sami) showed that the prevalence of general obesity (BMI ≥ 30 kg/m^2^) was higher in Sami compared with non-Sami, both in women (38·7 and 24·3%, respectively) and men (26·9 and 23·4%, respectively)^(^ ^24^ ^)^. Generally, there is little information about eating habits among Sami, although earlier studies have shown that Sami living in inland areas have reindeer meat as an important part of their diet and eat less fish and processed foods^(^ ^25^ ^)^. Interestingly, it is also in these areas that the obesity trends are most pronounced^(^ ^23^ ^,^ ^24^ ^)^. Comprehensive information on the health status and disease burden among various ethnic groups in northern Norway is lacking, and this is especially prominent within mental health issues. The Sami people have a long history of assimilation which likely has had an impact on mental health issues; however, it is unknown if this also affects DE.

Norway is characterized by universal public health insurance coverage and predominantly public health services which in general is a generous health service for the whole population. Nevertheless, the health services are also lacking sufficient health workers with competence in Sami language^(^ ^26^ ^)^. Several studies report that members of ethnic minorities suffering from an ED are less likely to seek help and, when they do, they are less likely to receive treatment^(^ ^27^ ^,^ ^28^ ^)^. From a clinical perspective, this may be important to reflect upon also in the Sami population.

In general, limited research exists on DE and ED among indigenous people even though many indigenous groups are at risk of under- and overweight. Mellor *et al*. showed that Aboriginal adolescents in Australia seem to be less dissatisfied with their body shape and weight compared with other Australian adolescents^(^ ^29^ ^)^. The view of what is an attractive appearance may also vary depending on cultural context. A study published in 2007 based on Sami, Finnish and British men showed a differential physical preference with regard to the female body^(^ ^30^ ^)^. While Sami men liked women with higher BMI and more ‘shapely’ bodies to a higher degree, urban Finnish and British men preferred slim bodies. Results from the North Norwegian Youth Study (1994–1995) found Sami boys to be more prone to various types of ED compared with non-Sami and Kven (a cultural minority of Finnish origin) boys, while Sami girls reported fewer eating problems, particularly bulimic ones, than the majority of girls^(^ ^31^ ^)^.

To our knowledge, no research related to ED or DE has been conducted in the adult Sami population. Increased awareness with regard to DE prevalence and associated risk factors in the Sami population could assist with preventive early intervention efforts in this population with increasing obesity problems^(^ ^24^ ^)^. Due to long-term marginalization and ‘Norwegianization’, factors known to affect DE such as pressures to attain body image ideals, emotional and mental health problems, and lack of appropriate health services may be elevated in the Sami. The main aim of the present study was therefore to investigate DE among Sami compared with non-Sami populations residing in the same geographical regions of Norway. Of DE traits, we presume BED-like symptoms to be most prevalent due to the quite high obesity prevalence in the target populations and the known association between BED and obesity.

## Methods

### The SAMINOR Study

The Population-based Study on Health and Living Conditions in Regions with Sami and Norwegian Populations – the SAMINOR Study – is the responsibility of the Centre for Sami Health Research at UiT The Arctic University of Norway. The overall purpose of the study is to produce knowledge on the health and living conditions of the indigenous Sami people in Norway. The first survey of the SAMINOR Study – SAMINOR 1^(^ ^32^ ^)^ – was conducted in 2003–2004 in collaboration with the Norwegian Institute of Public Health.

**Fig. 1:**
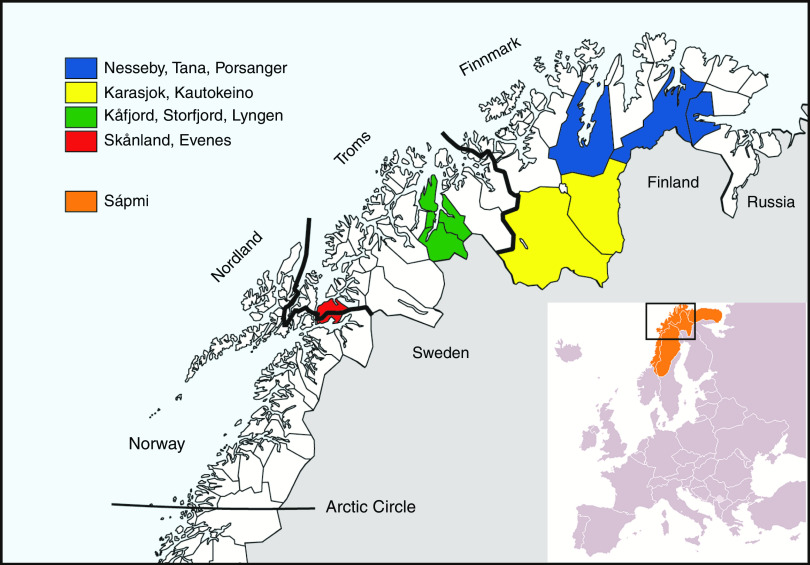
The four geographical regions included in the study and the municipalities within each. Sápmi is the cultural region traditionally inhabited by the Sami people. Sápmi is located in Northern Europe and includes the northern parts of Fennoscandia. The region stretches over four countries: Norway, Sweden, Finland and Russia

In 2012 the Centre for Sami Health Research initiated a follow-up survey, SAMINOR 2. The data collection was carried out in two parts. The first part was a questionnaire-based survey conducted in 2012 – The SAMINOR 2 Questionnaire Survey^(^ ^33^ ^)^. The second part – The SAMINOR 2 Clinical Survey – was conducted during 2012–2014. The clinical survey was conducted in ten selected municipalities, which all had been a part of the SAMINOR 1 survey. Inhabitants in the following municipalities were included: Karasjok, Kautokeino, Porsanger, Tana, Nesseby, Lyngen, Kåfjord, Storfjord, Skånland and Evenes (Fig. 1). Invitees were all residents in the age range 40–79 years. As no national records contain ethnic information, people were invited regardless of ethnic background.

The present analyses are based on cross-sectional data from the SAMINOR 2 Clinical Survey restricted to the ages 40–69 years due to the questionnaire design. In this age span there were 10 399 invitees. A total of 4876 individuals (2198 males and 2678 females) attended the clinical examination, a response rate of 46·9%. The response rate varied from 40·3% in Evenes to 54·4% in Kautokeino. The survey included an eight-page self-administered questionnaire with a more extensive FFQ than in the previous surveys. The questionnaire was provided in Norwegian and in some of the municipalities also in the Northern Sami language. The questionnaire (also a translated English version) is available online (http://www.saminor.no). Those who did not fill in the questionnaire (*n* 21) were removed. Further, we excluded a total of 405 participants who did not fill in all questions regarding DE and forty-three persons who did not fill in ethnicity information. Finally, eighteen persons who did not get their height and weight measured or who were pregnant, disabled or measured with shoes were excluded. After these exclusions, our study sample consisted of 4389 persons, 42·2% of the invited sample.

### Disordered eating

The Eating Disorder Examination (EDE) is an investigator-based interview and is widely viewed as the ‘gold standard’ measure of ED psychopathology^(^ ^34^ ^)^. Self-reported examinations such as EDE’s self-reported version, the EDE-Q with twenty-eight items^(^ ^35^ ^)^ and the self-reported EDI (Eating Disorder Inventory) with sixty-eight items have also been frequently used for assessment of AN and BN in clinical investigations^(^ ^36^ ^)^. With the aim of studying ED symptoms in community-derived samples, other self-report questionnaires with fewer items have been developed^(^ ^37^ ^–^ ^39^ ^)^ including the one used here: the Eating Disturbance Scale (EDS-5), which shows significant correlations with the EDI and even higher correlations with similar factors from the self-report version of the EDE^(^ ^38^ ^)^.

The EDS-5^(^ ^38^ ^)^ consists of the following five questions regarding eating habits within the past 4 weeks: (i) ‘Are you satisfied with your eating habits?’ (ii) ‘Have you eaten to comfort yourself because you were unhappy?’ (iii) ‘Have you felt guilty about eating?’ (iv) ‘Have you felt that it was necessary for you to use a strict diet or other eating rituals to control your eating?’ (v) ‘Have you felt that you are too fat?’ Each of these items was scored on a seven-point Likert scale ranging from 1 to 7 (most pathological response). The scores were summed to produce a total score ranging from 5 to 35. Persons who did not answer all five questions were excluded from the analysis. The sex-specific 90th percentile of the total sum score was used as cut-off to identify persons with a disturbed eating pattern, ≥19 for men and ≥23 for women.

In general, estimation of DE in the general population is challenging due to the very heterogeneous group and lack of well-suited instruments. EDS-5 has not been used frequently, which may be an important limitation to consider. Even so, the instrument was validated both at the initial construction^(^ ^38^ ^)^ and by Eik-Nes *et al*.^(^ ^15^ ^)^, who found a sum score ≥23 in a population-based sample of 16 412 women comparable to the mean sum scores in a clinical sample of sixty women diagnosed with severe ED.

### Ethnicity

Information on ethnicity was obtained through eleven questions regarding home language, ethnic background and self-perceived ethnicity/identity: ‘What language(s) do/did you, your parents and your grandparents use at home?’ The questions were to be answered separately for each relative. The response categories were ‘Norwegian’, ‘Sami’, ‘Kven’ or ‘Other’. Providing the same response options, we also asked: ‘What is your, your father’s and your mother’s ethnic background?’ The respondents also reported whether they considered themselves to be Norwegian, Sami, Kven or other (self-perceived ethnicity). On all these questions, multiple answers were allowed. Based on these questions, participants were categorized as Sami if they reported that they considered themselves to be Sami or that they had Sami ethnic background, and in addition reported Sami as home language for at least one grandparent, parent or themselves. All other participants were categorized as non-Sami.

### Anxiety and depression

Symptoms of anxiety and depression were assessed with the five-item Hopkins Symptom Checklist (SCL-5). The SCL-5 scale was developed and validated as a short version of the longer SCL-25 scale^(^ ^40^ ^)^; in a sample of 9380 participants from mid-Norway aged 40–42 and 65–67 years, a correlation of *r*=0·92 was found between SCL-5 and SCL-25. Another Norwegian sample of 7004 participants aged 16–97 years found a sensitivity of 82% and a specificity of 96% for SCL-5 with a cut-off value of 2·0, with SCL-25>1·75 as criterion^(^ ^41^ ^)^. Unfortunately, no validation studies have been performed on the Sami population. On a four-point Likert scale ranging from 1 (‘not bothered’) to 4 (‘very much bothered’), participants were asked about the following symptoms during the past 4 weeks: ‘nervousness or shakiness inside’, ‘feeling fearful’, ‘feeling hopeless about the future’, ‘worrying too much about things’ and ‘feeling blue’. An mean score of 2·0 or higher was used to identify anxiety or depression, following suggestions from Strand *et al*.^(^ ^41^ ^)^. The mean score was set to missing for participants who answered fewer than four of the questions (missing, *n* 191).

### Education, physical activity and dietary intake

Education was assessed by the question: ‘How many years of education have you completed? (Include any and all years in which you attended school or studied)’. Number of education years in the analyses was dichotomized into ‘13 years or more’ and ‘less than 13 years’.

Physical activity was assessed by self-report on an ordinal scale of 1 (very low) to 10 (very high). The scale has previously been validated for middle-aged women in Tromsø, Norway^(^ ^42^ ^)^, but has not been validated in men. The participants were informed in the questionnaire that ‘physical activity’ includes household chores and professional activities as well as regular exercise and other physical activity, such as walking/hiking. The physical activity levels were collapsed as follows: very low (levels 1 and 2), low (levels 3 and 4), moderate (levels 5 and 6), high (levels 7 and 8) and very high (levels 9 and 10).

A semi-quantitative FFQ was used to collect information on various food items consumed during the past year. The FFQ is based on the FFQ used in the Norwegian Women and Cancer Study (NOWAC). The NOWAC FFQ has previously been validated for the general female population of Norway and described in detail elsewhere^(^ ^43^ ^)^. Included in the FFQ were questions on how often various types of snacks (dark chocolate, milk chocolate, sweets/candy, potato crisps, peanuts, other nuts, other salty snacks) were consumed within the past year. Answering options were ‘never/rarely’, ‘1–3 times per month’, ‘once per week’, ‘2–3 times per week’, ‘4–6 times per week’ and ‘1+ times per day’. Missing answers were considered ‘no intake’. For dark chocolate and milk chocolate, the amount normally eaten each time was provided. For the other items, standard portions were used. Based on frequency and amount, the intake of each snack in grams per day was calculated. The total intake of snacks was obtained by summing grams per day for these seven variables. ‘Snacking’ was defined as having total snack consumption at or above the 90th percentile (37·8 g/d).

### Anthropometry

Height, weight and BMI were measured using an electronic height and weight scale (DS-103; Dongsahn Jenix, Seoul, Korea) with the participant wearing light clothing without shoes. Height was measured to the nearest 0·1 cm and weight to the nearest 100 g, and BMI=[weight (kg)]/height (m)]^2^ was calculated to the nearest 0·1 unit. According to the WHO, underweight was defined as BMI<18·5 kg/m^2^, normal weight as BMI=18·5–24·9 kg/m^2^, overweight as BMI=25·0–29·9 kg/m^2^, obesity class I as BMI=30·0–34·9 kg/m^2^ and obesity class II as BMI≥35·0 kg/m^2^.

### Statistics

All statistical analyses were performed using the statistical software package IBM SPSS Statistics for Windows, version 24.0. The analyses were performed separately for men and women.

Sample characteristics were treated as categorical variables and presented as numbers and percentage. Ethnic differences were tested using Pearson’s *χ* ^2^ tests (Table 1). When testing for differences in BMI, underweight participants (*n* 11) were combined with normal-weight participants due to low numbers.

**Table 1:** Characteristics of the sample of Sami and non-Sami Norwegian adults aged 40–69 years (*n* 4389*); SAMINOR 2 Clinical Survey (2012–2014)

	Men						Women					
	Sami (*n* 844*)		Non-Sami (*n* 1180*)		Total (*n* 2024*)		Sami (*n* 967*)		Non-Sami (*n* 1398*)		Total (*n* 2365*)	
	*n*	%	*n*	%	*n*	*%*	*n*	%	*n*	%	*n*	%
Age (years)												
40–49	210	24·9	297	25·2	507	25·0	287	29·7	426	30·5	713	30·1
50–59	275	32·6	370	31·4	645	31·9	347	35·9	455	32·5	802	33·9
60–69	359	42·5	513	43·5	872	43·1	333	34·4	517	37·0	850	35·9
*χ* ^2^(df), *P* value†	*χ* ^2^(2)=0·35, *P*=0·84						*χ* ^2^(2)=3·03, *P*=0·22					
Education (years)												
<13	494	60·4	651	56·7	1145	58·2	404	43·7	646	47·7	1050	46·1
≥13	324	39·6	498	43·3	822	41·8	521	56·3	707	52·3	1228	53·9
Missing	26		31		57		42		45		87	
*χ* ^2^(df), *P* value†	*χ* ^2^(1)=2·74, *P*=0·10						*χ* ^2^(1)=3·66, *P*=0·06					
BMI class												
Normal weight‡	169	20·0	215	18·2	384	19·0	273	28·2	502	35·9	775	32·8
Overweight	407	48·2	635	53·8	1042	51·5	390	40·3	550	39·3	940	39·7
Obese class I	214	25·4	250	21·2	464	22·9	209	21·6	258	18·5	467	19·7
Obese class II	54	6·4	80	6·8	134	6·6	95	9·8	88	6·3	183	7·7
*χ* ^2^(df), *P* value†,‡	*χ* ^2^(3)=7·67, *P*=0·05						*χ* ^2^(3)=22·51, *P*<0·001					
Anxiety and depression (SCL-5 mean score)												
<2·0	717	87·9	1051	92·6	1768	90·6	778	84·7	1189	89·5	1967	87·6
≥2·0	99	12·1	84	7·4	183	9·4	140	15·3	139	10·5	279	12·4
Missing	28		45		73		49		70		119	
*χ* ^2^(df), *P* value†	*χ* ^2^(1)=12·50, *P*<0·001						*χ* ^2^(1)=11·42, *P*=0·001					
Physical activity												
1–2	88	10·7	94	8·1	182	9·2	90	9·5	85	6·2	175	7·6
3–4	237	28·8	328	28·1	565	28·4	242	25·4	282	20·7	524	22·7
5–6	279	33·9	409	35·1	688	34·6	324	34·1	491	36·1	815	35·3
7–8	168	20·4	283	24·3	451	22·7	238	25·0	404	29·7	642	27·8
9–10	51	6·2	52	4·5	103	5·2	57	6·0	99	7·3	156	6·7
Missing	21		14		35		16		37		53	
*χ* ^2^(df), *P* value†	*χ* ^2^(4)=9·90, *P*=0·04						*χ* ^2^(4)=19·55, *P*=0·001					
Snacks§												
<37·8 g/d	782	92·7	1044	88·5	1826	90·2	880	91·0	1244	89·0	2124	89·8
≥37·8 g/d	62	7·3	136	11·5	198	9·8	87	9·0	154	11·0	241	10·2
*χ* ^2^(df), *P* value†	*χ* ^2^(1)=9·74, *P*=0·002						*χ* ^2^(1)=2·55, *P*=0·11					

SCL-5, five-item Hopkins Symptom Checklist.

* Subgroups may not total to this number due to missing values.

† Ethnic difference tested by Pearson’s *χ* ^2^ test.

‡ Due to few persons in the underweight category, these were combined with normal weight in the *χ* ^2^ tests.

§ Grams per day of dark chocolate, milk chocolate, sweets/candy, potato crisps, peanuts, other nuts, other salty snacks. Missing values are considered ‘no intake’.

The five questions that make up the EDS-5 were, together with the total score, presented as mean scores and 95% CI (Table 2). Ethnic differences were tested using two-sample *t* tests, assuming equal variances. Ethnic differences in the proportion above/below the 90th percentile of the EDS-5 score were tested using Pearson’s *χ* ^2^ tests.

**Table 2:** Items included in the EDS-5 score and total EDS-5 score, presented as means and 95% CI, together with dichotomized EDS-5 score presented as number and percentage above/below the 90th percentile, by sex and ethnic group, in the sample of Sami and non-Sami Norwegian adults aged 40–69 years (*n* 4389); SAMINOR 2 Clinical Survey (2012–2014)

	Men (*n* 2024)					Women (*n* 2365)				
	Sami (*n* 844)		Non-Sami (*n* 1180)			Sami (*n* 967)		Non-Sami (*n* 1398)		
	Mean	95% CI	Mean	95% CI	*t*(df), *P* value*	Mean	95% CI	Mean	95% CI	*t*(df), *P* value*
Eating habits	2·8	2·7, 2·9	2·8	2·7, 2·8	*t*(2022)=0·37, *P*=0·71	2·9	2·8, 3·0	2·9	2·9, 3·0	*t*(2363)=−0·73, *P*=0·48
Comfort eating	1·8	1·8, 1·9	1·7	1·7, 1·8	*t*(2022)=2·50, *P*=0·01	2·3	2·2, 2·4	2·1	2·0, 2·2	*t*(2363)=2·60, *P*=0·01
Felt guilty	1·8	1·8, 1·9	1·8	1·7, 1·9	*t*(2022)=1·07, *P*=0·28	2·4	2·3, 2·5	2·4	2·3, 2·5	*t*(2363)=−0·15, *P*=0·88
Eating rituals	2·0	1·9, 2·1	2·0	1·9, 2·1	*t*(2022)=0·57, *P*=0·57	2·5	2·4, 2·6	2·4	2·3, 2·5	*t*(2363)=1·09, *P*=0·28
Too fat	2·9	2·7, 3·0	2·9	2·8, 3·0	*t*(2022)=−0·45, *P*=0·65	3·7	3·6, 3·9	3·7	3·6, 3·8	*t*(2363)=0·47, *P*=0·64
EDS-5 score	11·3	11·0, 11·7	11·1	10·8, 11·4	*t*(2022)=0·94, *P*=0·35	13·8	13·4, 14·1	13·5	13·2, 13·9	*t*(2363)=0·86, *P*=0·39
	*n*	%	*n*	%	*χ* ^2^(df), *P* value†	*n*	%	*n*	%	*χ* ^2^(df), *P* value†
EDS-5 ≥19 (m)/≥23 (w)	104	12·3	128	10·8	*χ* ^2^(1)=1·06, *P*=0·30	104	10·8	139	9·9	*χ* ^2^(1)=0·41, *P*=0·52
EDS-5 <19 (m)/<23 (w)	740	87·7	1052	89·2		863	89·2	1259	90·1	

EDS-5, Eating Disturbance Scale; m, men; w, women.

* Ethnic difference tested by two-sample *t* test with equal variances assumed.

† Ethnic difference tested by Pearson’s *χ* ^2^ test.

To assess the effect various factors had on the EDS-5 score, the following predictors were included in our models: age, education, BMI class, anxiety and depression, physical activity and consumption of snacks. Stratified by ethnic groups, associations between the dichotomized EDS-5 score and the selected variables were presented as proportions and tested using the Mantel–Haenszel test for trend (linear-by-linear association; Table 3). We further explored these associations with logistic regression, adjusting for age and education. OR with 95% CI are presented (Table 4). *P* values less than 0·05 were considered statistically significant.

**Table 3:** Associations between dichotomized EDS-5 score and selected variables, presented as number and percentage above the 90th percentile cut-off (≥19 for men and ≥23 for women), by sex and ethnic group, in the sample of Sami and non-Sami Norwegian adults aged 40–69 years (*n* 4389*); SAMINOR 2 Clinical Survey (2012–2014)

	Men (*n* 2024*)				Women (*n* 2365*)			
	Sami (*n* 844*)		Non-Sami (*n* 1180*)		Sami (*n* 967*)		Non-Sami (*n* 1398*)	
	*n*	%	*n*	%	*n*	%	*n*	%
Total EDS-5 scoring	104	12·3	128	10·8	104	10·8	139	9·9
Age (years)								
40–49	36	17·1	41	13·8	41	14·3	52	12·2
50–59	34	12·4	39	10·5	35	10·1	46	10·1
60–69	34	9·5	48	9·4	28	8·4	41	7·9
*χ* ^2^(df), *P* value†	*χ* ^2^(1)=7·06, *P*=0·01		*χ* ^2^(1)=3·62, *P*=0·06		*χ* ^2^(1)=5·43, *P*=0·02		*χ* ^2^(1)=4·79, *P*=0·03	
Education (years)								
<13	74	15·0	61	9·4	45	11·1	65	10·1
≥13	28	8·6	64	12·9	55	10·6	70	9·9
*χ* ^2^(df), *P* value†	*χ* ^2^(1)=7·19, *P*=0·01		*χ* ^2^(1)=3·52, *P*=0·06		*χ* ^2^(1)=0·08, *P*=0·78		*χ* ^2^(1)=0·01, *P*=0·92	
BMI class								
Normal weight	2	1·2	4	1·9	4	1·5	10	2·0
Overweight	33	8·1	37	5·8	28	7·2	48	8·7
Obese class I	45	21·0	62	24·8	44	21·1	52	20·2
Obese class II	24	44·4	25	31·3	28	29·5	29	33·0
*χ* ^2^(df), *P* value†	*χ* ^2^(1)=84·2, *P*<0·001		*χ* ^2^(1)=103·5, *P*<0·001		*χ* ^2^(1)=83·7, *P*<0·001		*χ* ^2^(1)=114·7, *P*<0·001	
Anxiety and depression (SCL-5)								
<2·0	70	9·8	98	9·3	67	8·6	97	8·2
≥2·0	30	30·3	23	27·4	34	24·3	29	20·9
*χ* ^2^(df), *P* value†	*χ* ^2^(1)=34·1, *P*<0·001		*χ* ^2^(1)=26·6, *P*<0·001		*χ* ^2^(1)=29·7, *P*<0·001		*χ* ^2^(1)=62·4, *P*<0·001	
Physical activity								
1–2	13	14·8	20	21·3	12	13·3	14	16·5
3–4	46	19·4	53	16·2	34	14·0	44	15·6
5–6	25	9·0	33	8·1	36	11·1	44	9·0
7–8	15	8·9	18	6·4	16	6·7	24	5·9
9–10	3	5·9	3	5·8	5	8·8	7	7·1
*χ* ^2^(df), *P* value†	*χ* ^2^(1)=10·9, *P*=0·001		*χ* ^2^(1)=26·3, *P*<0·001		*χ* ^2^(1)=5·80, *P*=0·02		*χ* ^2^(1)=19·1, *P*<0·001	
Snacks								
<37·8 g/d	89	11·4	103	9·9	90	10·2	110	8·8
≥37·8 g/d	15	24·2	25	18·4	14	16·1	29	18·8
*χ* ^2^(df), *P* value†	*χ* ^2^(1)=8·72, *P*=0·003		*χ* ^2^(1)=9·02, *P*=0·003		*χ* ^2^(1)=2·83, *P*=0·09		*χ* ^2^(1)=15·3, *P*<0·001	

EDS-5, Eating Disturbance Scale; SCL-5, five-item Hopkins Symptom Checklist.

* Subgroups may not total to this number due to missing values.

† Mantel–Haenszel test for trend (linear-by-linear association).

**Table 4:** Age- and education-adjusted OR and 95% CI of associations between selected variables and dichotomized EDS-5 scores (≥19 for men and ≥23 for women), by ethnic group and sex, in the sample of Sami and non-Sami Norwegian adults aged 40–69 years (*n* 4389*); SAMINOR 2 Clinical Survey (2012–2014)

	Sami (*n* 1811*)				Non-Sami (*n* 2578*)			
	Men (*n* 844)		Women (*n* 967)		Men (*n* 1180)		Women (*n* 1398)	
	OR	95% CI	OR	95% CI	OR	95% CI	OR	95% CI
BMI class								
Normal weight	1·0	Ref.	1·0	Ref.	1·0	Ref.	1·0	Ref.
Overweight	7·4	1·7, 31·2	4·9	1·7, 14·4	3·2	1·1, 9·2	4·5	2·2, 9·0
Obese class I	20·8	4·9, 87·4	18·0	6·3, 51·5	17·9	6·3, 50·3	12·5	6·2, 25·2
Obese class II	63·1	14·1, 283·6	28·1	9·5, 83·4	25·4	8·4, 76·4	22·2	10·2, 48·2
Anxiety and depression (SCL-5)								
<2·0	1·0	Ref.	1·0	Ref.	1·0	Ref.	1·0	Ref.
≥2·0	3·7	2·2, 6·2	3·3	2·0, 5·3	3·5	2·1, 6·1	2·8	1·8, 4·5
Physical activity								
1–2	1·6	0·8, 3·3	1·1	0·5, 2·3	3·3	1·7, 6·1	2·0	1·0, 3·8
3–4	2·4	1·4, 4·0	1·2	0·7, 2·0	2·2	1·4, 3·6	1·8	1·2, 2·9
5–6	1·0	Ref.	1·0	Ref.	1·0	Ref.	1·0	Ref.
7–8	1·0	0·5, 1·9	0·6	0·3, 1·1	0·8	0·4, 1·4	0·6	0·3, 1·0
9–10	0·7	0·2, 2·4	0·7	0·3, 2·0	0·7	0·2, 2·4	0·7	0·3, 1·7
Snacks								
<37·8 g/d	1·0	Ref.	1·0	Ref.	1·0	Ref.	1·0	Ref.
≥37·8 g/d	2·6	1·3, 5·0	1·6	0·8, 3·0	1·9	1·1, 3·1	2·1	1·3, 3·4

EDS-5, Eating Disturbance Scale; SCL-5, five-item Hopkins Symptom Checklist; Ref., referent category.

* Subgroups may not total to this number, due to missing values.

## Results

### Descriptive statistics

Table 1 shows sex- and ethnic-specific characterization of the 4389 individuals (54% females) included in the study. Of these, 1811 individuals (41%) were defined as Sami (967 females, 844 males) and 2578 as non-Sami (1398 females, 1180 males). The age distribution in the three age groups (40–49 years, 50–59 years and 60–69 years) and the distribution between high and low education were well correlated among Sami and non-Sami participants.

The BMI-based weight distribution was significantly different between Sami and non-Sami women, with a higher proportion of Sami women *v*. non-Sami women defined as overweight (40·3 *v*. 39·3%, respectively) and obese (class I: 21·6 *v*. 18·5%; class II: 9·8 *v*. 7·7%, respectively). Compared with non-Sami men, the proportion of obese class I among Sami men was near significantly (*P*=0·05) higher (25·4 *v*. 21·2% in Sami *v*. non-Sami, respectively; Table 1).

### Disordered eating and associated factors

Mean EDS-5 scores were significantly higher in women compared with men, but there were no ethnic differences (mean score: 11·3 and 11·1 in Sami and non-Sami men, respectively; *v*. 13·8 and 13·5 in Sami and non-Sami women, respectively). Furthermore, there were no ethnic differences in the proportion at or above the 90th percentile, for either men or women. Moreover, across both sexes, Sami scored significantly higher on the ‘comfort eating’ item compared with the non-Sami: Sami men (mean score=1·8, 95% CI 1·8, 1·9) *v*. non-Sami men (mean score=1·7, 95% CI 1·7, 1·8) and Sami women (mean score=2·3, 95% CI 2·2, 2·4) *v*. non-Sami women (mean score=2·1, 95% CI 2·0, 2·2; Table 2).

Sami individuals were significantly more affected by anxiety and depression compared with non-Sami individuals (12·1 *v*. 7·4% in Sami *v*. non-Sami men, respectively; 15·3 *v*.10·5% in Sami *v*. non-Sami women, respectively; Table 1). In particular, the Sami responders scored higher than the non-Sami individuals on the SCL-5 item, ‘feeling hopeless about the future’. They also reported more anxiousness (data not shown).

With regard to physical activity, both Sami women and Sami men seemed in general to be less active than their non-Sami counterparts, although a higher proportion of Sami men scored at the highest physical activity level compared with non-Sami men (6·2 *v*. 4·5%, respectively). Sami men seemed to snack more (≥37·8 g/d) than non-Sami men (11·5 *v*. 7·3% in Sami *v*. non-Sami, respectively; Table 1).

Significant differences in DE were observed between age groups in Sami men (*P*=0·01) and women of both ethnic groups (*P*=0·02 and *P*=0·03 in Sami and non-Sami, respectively; Table 3), with higher percentages of DE with decreasing age. Education was significantly associated with DE only in Sami men, with higher education having a protective effect (*P*=0·01; Table 3).

In both Sami and non-Sami men and women, DE was associated with increased BMI (Tables 3 and 4). The odds of being categorized as having DE in obese class II individuals compared with normal-weight individuals were especially high for Sami men. Compared with non-Sami men, the associations between weight classes and EDS-5 scores were stronger for Sami men in all weight categories (Table 4). Similarly, an increased risk of suffering from DE in high weight categories also existed in women of both ethnic groups, although with a larger effect in Sami women in the two obese categories (Table 4).

Anxiety and depression was positively associated with disordered eating in all groups (Tables 3 and 4), with OR=3·7 (95% CI 2·2, 6·2) and OR=3·5 (95% CI 2·1, 6·1) in Sami and non-Sami men, respectively, and OR=3·3 (95% CI 2·0, 5·3) and OR=2·8 (95% CI 1·8, 4·5) in Sami and non-Sami women, respectively (Table 4).

There was a significant negative linear association between DE and levels of physical activity in both sexes and the two ethnic groups (Table 3). Further analyses showed that the odds of DE were double in the group exerting low physical activity compared with the moderately active group in men (OR=2·4, 95% CI 1·4, 4·0 in Sami *v*. OR=2·2, 95% CI 1·4, 3·6 in non-Sami, adjusted for age and education). In women, the association was significant in only non-Sami (OR=1·2, 95% CI 0·7, 2·0 in Sami *v*. OR=1·8, 95% CI 1·2, 2·9 in non-Sami; Table 4).

High degree of snacking (≥37·8 g/d) was significantly associated with DE in men (Sami and non-Sami), but for women only in the non-Sami group (Table 3). Sami men had more than doubled risk of DE when the degree of snacking was high compared with non-Sami men (OR=2·6, 95% CI 1·3, 5·0 in Sami *v*. OR=1·9, 95% CI 1·1, 3·1 in non-Sami, both adjusted for age and education). A similar effect was observed in women, but was significant only within the non-Sami group (OR=1·6, 95% CI 0·8, 3·0 in Sami *v*. OR=2·1, 95% CI 1·3, 3·4 in non-Sami, both adjusted for age and education; Table 4).

## Discussion

In our study we focused on DE in Sami and non-Sami populations residing in the same geographical areas in northern Norway. DE is of interest in our target population which suffers from increasing obesity prevalence. Additionally, increased awareness of DE and associated risk factors in the Sami population could assist in preventive and early intervention efforts in this population, which is also vulnerable due to long-term ‘Norwegianization’ and marginalization. We found no significant differences with regard to overall DE comparing the two ethic groups. However, DE in general was more prevalent in Sami men and the DE item, ‘comfort eating’, was reported more often in Sami compared with non-Sami. There were significant sex- and ethnic-specific associations between DE and physical activity, snacking and education level and in general. Furthermore, anxiety and depression and lower age were positively associated with DE.

The rates of overweight and obesity found in our overall study sample were even higher than found in other Norwegian populations, such as the Tromsø 6 (2007–2008)^(^ ^44^ ^)^ and the HUNT3 (2006–2008) survey^(^ ^45^ ^)^. By assuming a general continued obesity increase in Norway one would expect higher obesity prevalence today than measured 10 years ago and preliminary data from the Tromsø 7 (2015–2016) do show a continued increase from 2007–2008.

In our study, 12·3% of Sami men reported DE compared with 10·8% non-Sami men, while the rates were 10·8% in Sami women compared with 9·9% in non-Sami women. Both in our study and in another study where the same DE instrument and cut-off value were used^(^ ^15^ ^)^ lower prevalence was shown at increasing age. Individuals scoring high on DE in our study are not necessarily diagnosed with ED; however, they report signs of DE that may dispose to further increased risk of unhealthy weight-control behaviours and obesity. The DE instrument EDS-5 has been shown to be sensitive to DE patterns and appeared suited for screening purposes in community samples^(^ ^38^ ^)^. Even so, the EDS-5 instrument seems to better identify BN-^(^ ^46^ ^)^ and BED-related^(^ ^47^ ^)^ symptoms in community-based samples.

Studies addressing DE or ED in males are scarce, although the literature suggests a prevalence of 25% in community-based samples^(^ ^48^ ^)^. In our study men obtained a lower DE score compared with women and the 90th percentile showed a lower cut-off than in women (≥19 in men and ≥23 in women). Sami men had a higher proportion at or the above cut-off (12·3%) compared with non-Sami men (10·8%). The Sami population also scored significantly higher on the ‘comfort eating’ item compared with the non-Sami population in both sexes. Food preference and intake depend on complex mechanisms which involve the hedonic/pleasure component of eating (liking) and the incentive to eat (‘wanting’ or ‘reward-seeking’)^(^ ^49^ ^)^. Comfort eating (i.e. eating because of feeling worried or upset and not because of hunger) may occur when palatable food is available and as a response to stress, providing a potential means for people to ‘self-medicate’ for stress relief^(^ ^50^ ^)^. Regardless of ethnic group, palatable food is equally available throughout Norwegian society.

Emotional eating, or the tendency to eat in response to stress and emotions, is associated with overweight and obesity^(^ ^51^ ^,^ ^52^ ^)^. Depression is a strong predictor of dietary quality and BMI^(^ ^53^ ^)^, and findings from a Finnish study suggest that emotional eating and depressive symptoms affect unhealthy food choices^(^ ^54^ ^)^ such as consumption of snacks. Sex differences linked to psychological eating-related behaviours have been acknowledged, although little research is available focusing on this issue. In a recent study, sex differences in psychological predictors of snacking among a large representative Dutch sample were investigated. Although results suggested women to consume healthier foods in general and eat less unhealthy snacks than men, the relationships between psychological eating-related variables and snack intake were found to be similar for men and women^(^ ^55^ ^)^. In our study we observed snacking to be significantly associated with DE in men in general. In women, however, snacking was significantly associated with DE only in the non-Sami group.

Our findings suggest a strong association between weight problems and DE in both sexes and ethnic groups. Compared with normal-weight individuals, overweight and obese individuals had higher odds of DE in both sexes. The associations between DE and BMI were stronger in the obese and extreme obese categories, with OR as high as 63·1 (Sami men), 25·4 (non-Sami men), 28·1 (Sami women) and 22·2 (non-Sami women) in the obese class II category (BMI≥35 kg/m^2^). This finding agrees with the study on DE in the Norwegian HUNT population, which identified an OR ratio of 22·5 for women in the comparable obesity category (class II)^(^ ^15^ ^)^. In our study, the OR were higher in the Sami compared with the non-Sami population, but the CI were larger, indicating more inaccurate effect sizes.

A higher socio-economic status (measured by education level) was in our study significantly protective against DE only in Sami men. The lack of an association between DE and social status in the other groups is in agreement with recent findings^(^ ^56^ ^,^ ^57^ ^)^.

Our analyses showed that the risk of DE was approximately doubled in the group of men exerting low physical activity compared with the moderately active group. For women, an increased effect of low physical activity was observed only in the non-Sami group. Excessive exercise is in general a persistent behaviour in the more classically defined ED and is believed to be implicated in the aetiology, development and maintenance of these disorders^(^ ^58^ ^)^. Earlier findings suggest that individuals with ED may exercise to control body weight and shape, which consequentially improves their self-esteem^(^ ^59^ ^)^.

There were indications of protective effects with regard to DE at high physical activity levels, although these associations were not found to be significant. Within the ED literature, excessively driven exercise behaviours are most often conceptualized as compulsive^(^ ^60^ ^)^. However, the individuals scoring high on DE symptoms in our study are not patients and may be representing individuals more similar to the DE category classified with binge eating and bulimic symptoms.

The consequences of the ‘Norwegianization’ and marginalization that have occurred over so many years have not been fully investigated in the Sami population. Owing to this we are not aware of all the health-related consequences this may have had. In the rural society in northern Norway, both Sami- and non-Sami, there is very rapid development with an increasing pressure to accommodate to a ‘9-to-5’ lifestyle. There are fewer jobs in the primary industries to which reindeer husbandry belongs, and the alternatives are either education or unemployment^(^ ^61^ ^,^ ^62^ ^)^. Within the Sami communities this development has led to the women leaving due to education opportunities elsewhere^(^ ^63^ ^)^ while men stay in their home regions. This is also a trend in other indigenous societies^(^ ^64^ ^,^ ^65^ ^)^.

Some of our findings could reflect issues in the context considered above and could be a sign of a changing society where neither the health service nor the other societal establishments have managed to support the population to handle the societal changes. In Sami men, more DE was reported as well as stronger associations between DE and lower education, lower physical activity and higher consumption of snacks. These discoveries could reflect the growing differential roles of Sami men and women which urge further emphasis and preventive measures.

### Strengths and limitations

The SAMINOR Study is the only large population-based study especially designed to investigate the health of the Sami people in Norway. The SAMINOR 2 Clinical Survey was performed in municipalities with a large proportion of Sami inhabitants in close collaboration with local health authorities. Individual information on ethnic background enabled us to compare Sami and non-Sami living in the same geographical regions. Due to a cross-sectional design, assessing potential causal relationships due to temporal bias was not possible.

A strength of the present study was that the anthropometric clinical measurements were measured electronically by trained personnel, avoiding the pitfall of under- or misreporting weight-related measures^(^ ^66^ ^)^.

The participation rate in the study was 47%, which we consider satisfactory. In each municipality, data collection was carried out within only 2–7 weeks, which limited the invitees’ opportunity to attend. Participation was higher for women than for men, and increased with age. The results are therefore more uncertain for men and in general for the younger age groups. As registration of ethnic affiliation is prohibited in public records, we do not know whether the ethnic distribution in our sample reflects that of the targeted population. However, the highest participation rates were observed in the Sami-dominated municipalities of Kautokeino, Tana and Nesseby in Finnmark, which suggest a higher participation rate among the Sami in these areas. This also affects the composition of different Sami subgroups in the sample. Our sample is over-represented by Sami from Finnmark and correspondingly under-represented by Sami residing further south.

A limitation of our study is the high age of the participants (40–69 years), as ED overall are more prevalent at younger age^(^ ^6^ ^)^. Age-related effects appear not so robust for BED^(^ ^6^ ^)^ and as the EDS-5 seems to comply best with BN and BED^(^ ^47^ ^)^, age may not be of major importance here. In our study DE was more prevalent in the younger age groups although it was also quite high in the older age groups as well. Limited research exists on ED among mid-life individuals yet studies show ED not to be uncommon and the mean age of individuals reporting ED behaviours is relatively high^(^ ^67^ ^,^ ^68^ ^)^, making it reasonable to focus on these age groups.

Our ethnic definition is based on self-report. To be categorized as Sami, participants had to report that they considered themselves to be Sami or that they had Sami ethnic background. By additionally requiring Sami as a home language in at least one person in the past three generations, we ensured an objective connection to the Sami. Our ethnic definitions have limitations and may have different validity for different geographic regions and subgroups of the Sami population. The diversity within the Sami group with regard to cultural identity and mode of living may also have diluted the ethnic-specific effects observed in our study.

## Conclusion

The main aim of the present study was to investigate DE among Sami compared with non-Sami populations residing in the same geographical regions of northern Norway. We found no overall significant differences with regard to overall DE comparing the two ethnic groups. However, DE was reported more often in Sami men and the DE item, ‘comfort eating’, was reported more often in Sami individuals. There were significant sex- and ethnic-specific associations between DE and physical activity, snacking and education level. In general, anxiety and depression and lower age were positively associated with DE.
